# Mitochondrial DNA Mutations in etiopathogenesis of male infertility

**DOI:** 10.4103/0970-1591.40606

**Published:** 2008

**Authors:** Monis Bilal Shamsi, Rakesh Kumar, Audesh Bhatt, R. N. K. Bamezai, Rajeev Kumar, Narmada P. Gupta, T. K. Das, Rima Dada

**Affiliations:** Laboratory for Molecular Reproduction and Genetics, Department of Anatomy, AIIMS, New Delhi, India; 1 National Centre for Applied Human Genetics, School of Life Sciences, JNU, New Delhi, India; 2 Department of Urology, AIIMS, New Delhi, India; 3 Electron Microscope Facility, Department of Anatomy, AIIMS, New Delhi, India

**Keywords:** Assisted reproduction techniques, asthenozoospermia, infertility, Mitochondrion and mitochondrial DNA mutations, mt genome

## Abstract

**Objective:**

To understand role of mitochondrial (mt) mutations in genes regulating oxidative phosphorylation (OXPHOS) in pathogenesis of male infertility. Infertility affects approximately 15% of couples trying to conceive. Infertility is frequently attributed to defects of sperm motility and number. Mitochondrion and mitochondrial DNA (mtDNA) play an important role in variety of physiological process. They control the oxidative energy supply and thus are central to growth, development and differentiation. Mitochondrial function is controlled by a fine-tuned crosstalk between mtDNA and nuclear DNA (nDNA). As mitochondria supply energy by OXPHOS, any mutation in mtDNA disrupts adenosine triphosphate (ATP) production and thus result in an impaired spermatogenesis and impaired flagellar movement. As sperm midpiece has few mtDNA copies, thus enhanced number of mutant mtDNA results in early phenotypic defect which manifest as spermatogenic arrest or asthenozoospermia. Oxidative stress and mtDNA mutations are positively correlated and mutations in mitochondrial genome (mt genome) are implicated in the lowered fertilising capacity of the sperm and affects the reproductive potential of an individual.

**Materials and Methods:**

A thorough review of articles in the last 15 years was cited with reference to the below-mentioned keywords. The articles considered discuss the role of mt genome in the normal functioning of sperm and the factors associated with mt mutations and impact of these mutations on the reproductive potential.

**Results:**

Sperm motility is a very important factor for the fertilisation of ova. The energy requirements of sperm are therefore very critical for sperm. Mutations in the mitochondrial genes as COX II, ATPase 6 and 8 play an important role and disrupts ATP production affecting the spermatogenesis and sperm motility. Therefore, the aberrations in mt genome are an important etiopatholgy of male infertility.

**Conclusion:**

In the context of male infertility, mt mutations, generation of reactive oxygen species and lowered antioxidant capacity are interlinked and constitute a unified pathogenic molecular mechanism. In the era of assisted reproduction technique (ART), it is very important to distinguish between mutations in nuclear and mitochondrial genomes in sperm, as mtDNA mutations are better diagnostic and prognostic markers in infertile men opting for ART.

## INTRODUCTION

Approximately, 30-40% men in reproductive age group have qualitative or quantitative defect in the sperm production. In about 50% of these cases, infertility can be attributed to low sperm motility (asthenozoospermia) or/and low sperm count (oligospermia). [1] Mutation and depletion of mitochondrial DNA (mtDNA) are associated with poor motility and diminished fertility of human sperm. The mtDNA is linked to electron transport system and is thus vulnerable to free radical damage and some of its components mutate 100 times more rapidly then nuclear DNA (nDNA). The risk of reactive oxygen species (ROS) induced damage is so high that no oxidative phosphorylation (OXPHOS) occurs in nucleus. The process occurs in cytoplasmic organelles as mitochondrion which are reduced to disposable elements in the sperms following fertilisation. [2]

Sperms require greater amount of energy for their survival and proper functioning, so sperm mitochondrion are uniquely placed in the midpiece to provide energy quickly and effectively for sperm motility. [3] During the maturation of sperm there is a shift in the pattern of adenosine triphosphate (ATP) utilisation, initially for biosynthesis and in later stages for motility. [4] In the early phases of fertilisation, sperms require ATP for the flagellar movement. [5] This rigorous energy demand is facilitated by mitochondrion through OXPHOS via the electron transport chain (ETC). The ATP generated in mitochondrion is transported to the motility apparatus of the sperm tail via the creatine phosphate shuttle or by vertical or radial diffusion. [6–9]

The electrochemical gradient generated by the transfer of protons from inside the mitochondrial matrix across the inner membrane into the intermembrane space serves as a source of potential energy for synthesising ATP by complex V. [10]

The proteins coded by both the nuclear genomes (nDNA) and mitochondrial genome (mtDNA) are vital for the effective reproductive potential of sperm. However, the full extent of relationship between the status of mtDNA and nDNA in either somatic cells or sperm DNA has not yet been determined.

During spermatogenesis, the mitochondria undergo drastic morphological changes and subcellular reorganisation. [11] A reduction in the number of mitochondria and of the mitochondrial genome has been observed in the maturation of mammalian sperm. [12] Since the bioenergetic function of mitochondria is crucial for sperm motility, any quantitative or qualitative aberrations in mtDNA effects the cellular functioning of the spermatozoa. In sperms, specific mt deletions are associated with inadequate sperm functions. Multiple deletions of 7345 and 7599 bp mtDNA are associated with poor sperm motility. [13,14] Oligoasthenozoospermia in many cases is related with multiple mtDNA rearrangements. [15]

## METHODOLOGY

Articles related to the role of mitochondria in male fertility and impact on reproductive potential of sperm due to mt mutations were reviewed. The relationship between the oxidative stress and mt mutations was also analysed in the articles considered. A thorough review of literature from Pubmed Central (PMC) Proceedings of the National Academy of sciences (PNAS) on the related topic was done. The articles during the last 15 years were searched mainly and the search was in reference to the mentioned keywords. A total of 63 articles were short listed, considering the relevancy only 47 articles for taken into consideration for this review. The included articles correlated the mtDNA, mtDNA mutations and the reproductive potential of an individual. The articles discussing the association of ROS with mt mutations; the mt functioning; the mitochondrial and nuclear genome correlations were also considered.

Both indexed and non-indexed journals for the related topics were screened. Our own reported and published findings have also been included in the study.

## MITOCHONDRIAL FUNCTIONING

Mitochondrion is assembled from proteins derived from about 1000 genes derived from mitochondrial and nuclear genome. The enzymes for the energy production OXPHOS are located at the inner mitochondrial membrane. OXPHOS is composed of five multipolypeptide enzyme complexes. Complexes I, II, III and IV constitute the ETC while the complex V is the ATP synthetase. The oxidation of metabolites by tricarboxylic acid cycle (TCA) and the β-oxidation pathways liberates CO_2_ and hydrogen atoms, the latter being transferred to soluble NAD^+^ to generate NADH or to enzyme bound FAD^+^ to yield FADH. [2] Complexes I-IV involved in the ETC generate electrochemical gradient by the transport of protons into the intermembrane space through the controlled oxidation of the electrons. This electro-potential difference serves as a source of potential energy for synthesising ATP which is generated in the complex V (ATP synthase). Mitochondrial functioning is controlled by complex cross talk between nuclear and mitochondrial genomes.

## MITOCHONDRIAL GENOME

About 70-80 mitochondria are present in the midpiece of a single mammalian sperm. [16,17] Mitochondrion is the only organelle in the sperm which has its own genome: mtDNA. The existence of this extra chromosomal genetic system has conferred mitochondrion with the ability to synthesise proteins in a semi-autonomous manner. [18] Human mtDNA is a 16569 bp double stranded circular DNA molecule coding for 2 rRNAs, 22 tRNAs and 13 polypeptides. [19] During the course of evolution most mt genes have migrated to the nucleus and only genes regulating OXPHOS pathway have remained on mtDNA. This reduction in genomic material is extreme leaving little room for error. Since the mitochondrial genome consists of no introns but only exons, every point mutation or deletion has the capacity to effect the mitochondrial function of cellular respiration support. Mitochondrial DNA also lacks the protection of histones or DNA-binding proteins and is believed to have only a very basic repair mechanism. Moreover, the mitochondrial genome is attached to the inner membrane of mitochondrion where ROS and free radicals (e.g., ubisemiquinone radicals) are continually generated by the respiratory chain. This basic repair mechanism can repair excision and base mutation caused by oxidative insult. The mutation rate is 10-100 times higher than nDNA because of the ROS-induced damage, higher replication rate, absence of a significant proof reading system and the cumulative damage from the free radicals generated by the electron chain. Intergenerational passage of mtDNA rests on a quantitative restriction event “Bottle neck” which restricts available genotypes and produces founder effect, a massive amplification of copy number during which genetic drift occurs.

The replication of mtDNA and gene expression of the mitochondrial genome are executed and regulated by enzymes and proteins factors encoded by nDNA. Human mtDNA is maternally inherited and co-existence of mutant DNA molecules with wild-type mtDNA in the same cell, i.e., hetroplasmy is also observed, providing a mosaic of mutation load with in the same sperm cell. [20] Proper nucleocytoplasmic ratio is required to support normal chromosomal segregation and effects normal embryo development potential. Though paternal DNA is eliminated in species-specific manner post-fertilisation during embryogenesis. Few recent studies have identified paternal mtDNA in ART conceived embryos. However, the survival is unpredictable and erratic. Thus the mitochondrial health of the embryo is critical for normal embryonic development and has lifelong implications on health.

## MITOCHONDRIAL MUTATIONS

Mitochondrial dysfunction is associated with a number of bioenergetic disorders ranging from skeletal to cardiomyopathies and neuromyopathies. Large-scale mtDNA deletions cause chronic progressive external opthalmoplegia (CPEO), Kearns-Sayre syndrome (KSS) and Pearsons marrow-Pancreas syndrome. The most common mtDNA mutation affecting 40% of patients with mitochondrial myopathy is the 4977 bp deletion occurring at the break points of the 13 bp direct repeats of 5´-ACCTCCCTCACCA-3´.[20,21]

Mitochondrial DNA mutations identified in several studies have been associated with poor semen quality. Two specific point mutations in the region of mtDNA genome from COX I (complex IV) and ND 5 (complex I), at nt 9055 and nt 11719 are associated with poor semen quality parameters in 11 and 12%, respectively, of cases. [22,23] Sperm mtDNA is highly vulnerable to mutations due to increased ROS and free radicals produced as byproducts during aerobic respiration. This peroxidative damage is also correlated with the decrease in the antioxidant enzyme level. Age-related increase in oxidative stress and oxidative damage to mtDNA is also implicated in male infertility. [24] The most common deletion in human sperm mtDNA is the 4977 bp deletion [13] occurring between the two replications origins of mtDNA. Mitochondrial DNA is commonly mutated during the process of spermatogenesis. The three-strand intermediate formed during the mtdNA replication is highly susceptible to the large-scale deletions via slippage mispairing. At this stage the mt genome is also highly exposed to ROS and mutations may be induced if intracellular levels of antioxidants are insufficient for protection of spermatozoa from oxidative damage. [24,25]

Both qualitative (e.g., mutation) and quantitative changes (e.g., reduction in the copy number) of mtDNA are observed in the affected tissues of patients having mitochondrial disorders. Gene(s) responsible for mtDNA depletion are encoded by the nuclear genome.

### Mitochondrial Mutations and Sperm Differentiation

Mammalian spermatogenesis is affected by any defect in mitochondrial respiratory activity especially the progression of the pachytene stages during meiosis and sperm formation. When large amounts of pathogenic mutant mtDNA accumulates in the testes, mitochondrial respiratory dysfunction is induced in spermatogenic cells. The reduction of energy production by the mitochondrion induces meiotic arrest during spermatogenesis. The respiration-deficient spermatocytes may not complete meiosis and these cells are removed by apoptosis. [26–28] Spermatocytes having lower mutated mtdNA load complete meiosis and transform into haploid spermatids. These spermatids could differentiate into sperm, but most of them show lower COX activity and abnormalities in the midpiece and nucleus. Therefore, oligospermia and asthenozoospermia are induced by meiotic arrest and enhanced apoptosis during spermatogenesis and the generation of sperms with mitochondrial respiration deficiency and abnormal morphology, respectively, resulting in male infertility. [29]

### Abnormal Mitochondrion and Infertility

Sperm motility is a major determinant of male fertility. Sperms with poor motility cannot penetrate through the mucus filled cervix to reach the site of fertilisation. Hence active flagellar motion is essential for successful fertilisation. Factors such as genital infections, maturational abnormalities in the epididymis, defects in flagellar axonemes or defects in metabolism cause impairment of sperm motility leading to infertility. [30] Absence or abnormal mitochondrion has been reported in asthenozoospermic men. [31–33] Oxidative stress is a common cause for mtDNA mutations.

**Figure 1:**
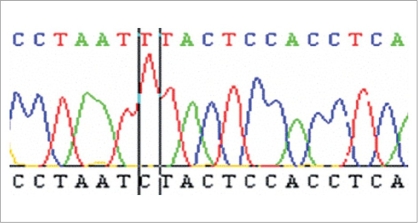
Mutational change from T to C at nucleotide position 12705 in ND5 gene in a case with OAT

**Figure 2:**
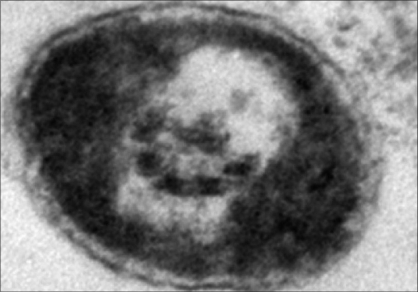
TEM picture shows partially formed microtubular appratus in sperm principal piece ×15000

In recent study by Kumar and associates reported mtDNA mutations in men with udiopathic infertility. [34] These men were cytogenetically normal and did not harbour Yq microdeletion spanning the azoospermia factor loci. [34] In the 49 oligospermic cases screened, 23 cases with oligoasthenozoospermia had mt mutation in the OXPHOS pathway. The mt mutation analysis of the 49 asthenospermic cases revealed deletions in the ATPase 6, ATPase 8 and COII gene. Out of 22 nucleotide substitutions observed in the 23 asthenospermic cases, 9 were in ATPase 6, 6 were in ATPase 8 and 7 were in COII. Of the 22 nucleotide changes, 5 were mis-sense mutations and 17 were silent mutations. Among these 23 oligoasthenozoospermic men an A > G transition at nucleotide 8860 was found in 20 patients and another transition A > G at nucleotide 8701 was detected in 12 cases. Figure 1 shows mutational change from T to C at nuclotide postion 12705 in ND5 gene in single case with oligoasthenoteratozoospermia (OAT). In 7 of these 12 cases, the sperms had axonemal defect with incomplete microtubular assembly [Figure 2]. Of these, six were novel polymorphisms in the mitochondrial genome present in almost 50% of the cases. This amino acid change from threonine to alanine could result in defective protein production. The ATPase 6 protein is one of the seven proteins that make up the Fo subunit of the mitochondrial FoF1-ATPase synthase (complex V) of the ETC, hence mutations in the mitochondrial genome have been shown to be associated with the poor semen parameters such as impaired sperm motility and incomplete maturation, the overall impact is the compromised reproductive potential of the male gamete. [34] Recent studies have reported that sperms with impaired motility have a higher copy number (higher mtDNA per cell) as compared to progressively motile sperms. It is believed that these are mtDNA with deletions which replicate faster than mtDNA.

## DISCUSSION

Mitochondrial DNA mutations play a major role in etiopathology of male infertility.

Mitochondrial DNA codes for genes which are required in OXPHOS pathway for ATP synthesis. ATP is required both for biosynthesis during spermiogenesis and for motility during fertilisation. [1]

The large-scale deletions and several mutations in COX II, ATPase 6 and 8 play an important role and disrupt ATP production and thus the partial and complete spermatogenesis arrest and impaired sperm motility. Since sperms require a substantial amount of energy to swim fast enough to reach the oviduct during fertilisation, the appropriate bioenergetic function of mitochondrion is crucial for male fertility. [5] About 5% of the O_2_ utilised by mitochondrion is converted to superoxide anions and other ROS in the active respiration state. The ROS in their balanced concentration play an important role in the capacitation of sperms. The ROS is also implicated in gene regulation, notably up-regulation of anti-oxidant proteins during higher oxidative stress and also have a pro-apoptotic effect. Superoxide anions are converted to hydrogen peroxide by the enzymatic action of superoxide dismutase. H_2_O_2_ thus produced leads to the generation of hydroxyl radicals. Mitochondrial DNA, attached transiently to the inner mitochondrial membrane is thus extremely vulnerable to oxidative damage under active respiration of spermatozoa. [35] Mitochondrial DNA is extensively damaged by ROS and this induces DNA strand breaks and large-scale deletions. The large-scale deletions result in complete removal or truncation of some structural and tRNA genes of mtDNA. The defective protein subunits encoded by such mutated mtDNA when assembled with nuclear encoded subunits yield impaired respiratory enzymes. Spermatozoa containing defective mitochondrion not only produce ATP less efficiently, but also generate more ROS and free radicals, which may further damage mitochondrion and mtDNA leading to an ultimate energy crisis and decline of motility and fertility.

Use of sperms with abnormal or dysfunctional spermatozoa in intracytoplasmic sperm injection may result in impaired recognition of mutant mtDNA and thus the transmissions of abnormal paternal DNA which is detrimental to embryonic growth. Thus mtDNA in male germ cells have suffer repetitive damage as it undergoes several waves of mitosis and the mature sperm relies heavily on mt electron transport chain to propel it into egg during fertilisation. Nature has evolved elaborate mechanisms to exclude paternal mtDNA from mt genome of the embryo.

Nuclear gene defects may also result in mitochondrial disorders by predisposing the cell to multiple mtDNA deletions. Hence mitochondrial respiratory chain function depends on the coordinated gene expression of both the mitochondrial and nuclear genomes. A mutation in either genome may lead to defective respiratory function of mitochondrion.

Moreover, mutated mtDNA molecules may be propagated by defective nuclear factors from paternal or maternal germ cells, be clonally expanded during the maturation of oocytes and be localised in primordial germ cytoplasm during embryogenesis. The oocytes ATP content (dependent on normal mtDNA) correlates with development potential of embryo after fertilisation. The change of sperm mtDNA copy number is very important event throughout spermatogenesis, fertilisation and embryogenesis. However, over reduction in the number of sperm mtDNA during or after spermatogenesis is detrimental to spermatozoa. Depletion of sperm mitochondrion is an important contributory factor in diminished sperm motility and fertility.

## CONCLUSION

mtDNA do play an important role in the spermatogenic arrest and decline of various motility parameters that are important determinants of male fertility. However, such cases which harbour mt mutations have a better prognosis on ART because paternal mitochondria are not transmitted to the offspring while mutations in the nuclear genome or cytogenetic anomalies may be iatrogenically transmitted. Thus, it is important to distinguish the type of genetic anomaly in infertile males, as this aids in providing the most adapted therapeutics to the infertile couples.
